# Permanent Catheter Placement for Recurrent Pericardial Effusions in the Presence of Dense Adhesions and Loculations

**DOI:** 10.7759/cureus.101585

**Published:** 2026-01-15

**Authors:** Steven Pong, Herbert Downton Ramos, Kriti Mittal, Justin Van Backer

**Affiliations:** 1 Surgery, Frank H. Netter MD School of Medicine, North Haven, USA; 2 General Surgery, University of Connecticut School of Medicine, Farmington, USA; 3 Division of General Thoracic Surgery, Hartford Healthcare, Hartford, USA

**Keywords:** pericardiac effusion, pericardial drainage, pericardial window, permanent indwelling catheter, pleurx catheter

## Abstract

Pericardial effusion (PcE) represents a spectrum of clinical presentations, ranging from incidental findings to life-threatening cardiac tamponade. While pericardiocentesis and surgical pericardial window are considered typical management approaches, recurrent and refractory cases in medically complex patients present unique challenges. Permanent indwelling catheters (PiCs), which are widely used for pleural effusions, have rarely been applied in the pericardial space. We describe the case of a 36-year-old woman with cerebral palsy, Pierre Robin syndrome, and a history of recurrent PcE and pleural effusions after failed pericardiocentesis and pericardial window. Given her complicated anatomy and comorbidities, a video-assisted thoracic surgery pericardial window with PiC placement was attempted. The effusion was heavily loculated and viscous, limiting catheter efficacy; drainage remained minimal, and the device was removed one month later. This case highlights both the potential role and limitations of PiCs for managing recurrent or refractory PcE.

## Introduction

Pericardial effusion (PcE) is the pathological accumulation of fluid within the pericardial sac, which normally contains only 10-50 mL of serous fluid [1]. Acute effusions can rapidly exceed the pericardium’s capacity, leading to cardiac tamponade, a life-threatening condition characterized by decreased cardiac output due to compression of the heart, leading to reduced preload. Chronic effusions allow for pericardial stretching but continue to carry the risk of progression to tamponade.

Echocardiography is the primary diagnostic tool, allowing real-time visualization of pericardial fluid, assessment of effusion size and distribution, and detection of hemodynamic consequences such as chamber collapse and respiratory variation in Doppler flows. When echocardiography is inconclusive or further anatomic detail is requested, CT and cardiac magnetic resonance offer higher fidelity.

Management of pericardial effusions typically follows a stepwise approach depending on effusion size, clinical symptoms, and underlying etiology [2,3]. Drainage is indicated in the setting of cardiac tamponade, suspected bacterial or neoplastic etiology, or persistent moderate-to-large effusions of unclear cause [4]. Most patients undergo pericardiocentesis unless the effusion is inaccessible, loculated, or associated with conditions that require surgical intervention, in which case a pericardial window is performed. In refractory, recurrent cases, a pericardiectomy may be considered. After drainage, fluid is routinely analyzed, and pericardial tissue sampling is reserved for cases in which malignancy, tuberculosis, purulent pericarditis, or recurrent effusion necessitate definitive evaluation. Patients without a clear etiology or indication for further intervention are generally managed with serial clinical and echocardiographic follow-up.

Permanent indwelling catheters (PiCs) are well established for managing recurrent pleural effusions, yet their use within the pericardial space is rarely described and off-label. To date, two cases have been reported: one in a heart transplant recipient and one in a patient with malignant effusion, both of which were free-flowing and achieved durable palliation. We present a unique case of recurrent, loculated PcE in a medically complex patient in whom placement of a PiC was attempted after multiple failed interventions. The case highlights both the potential role and limitations of this novel strategy.

This case report, in part, was previously presented as an oral presentation at the 2024 Connecticut Chapter of the American College of Surgeons Professional Association Annual Meeting (October 11, 2024, Trumbull, Connecticut).

## Case presentation

A 36-year-old woman with cerebral palsy, Pierre Robin syndrome, non-verbal status, and G-tube dependency, residing in a skilled nursing facility, had a history of recurrent pericardial and pleural effusions after failed pericardiocentesis and pericardial window through an anterolateral thoracotomy. The prior pericardiocentesis was complicated by a right ventricular perforation, although the catheter was removed in the operating room without further sequelae. The pericardial window was performed two months earlier. She presented with worsening shortness of breath, tachycardia, and hypotension. Given the large recurrent effusion, it was believed to be contributing to her respiratory compromise, and due to poor hemodynamics, she could not be safely diuresed. Rheumatology and infectious disease were consulted, and the workup was negative.

**Figure 1:**
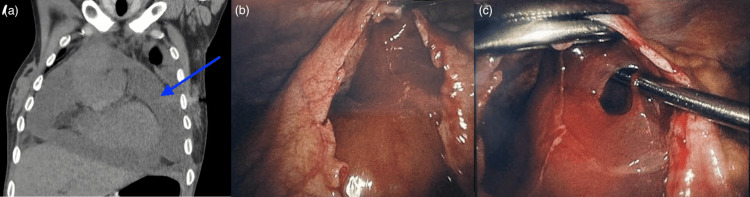
CT scan demonstrating a moderate-to-large pericardial effusion and intraoperative views showing loculations and adhesions. (A) Coronal chest CT image with blue arrow indicating a large pericardial effusion. (B, C) Intraoperative views through the pericardial window demonstrating thick, loculated pericardial fluid and dense adhesions between the pericardium and myocardium.

CT scan showed a moderate-to-large PcE along the right ventricular free wall and right atrium, but no cardiac tamponade (Figure 1A). Hounsfield unit measurements across multiple loculated regions of the effusion were approximately 15, suggestive of low-attenuation, serous fluid. Bilateral pleural effusions and a normal biventricular ejection fraction were noted on echocardiography. Due to this challenging case and failed previous medical and surgical treatments, a multidisciplinary discussion was held between thoracic surgery, cardiology, and cardiac surgery. The decision was made to attempt a video-assisted thoracic surgery (VATS) pericardial window and placement of a PleurX, a PiC (PleurX™, Becton, Dickinson and Company, Franklin Lakes, NJ, USA) in the pericardium. She underwent a right-sided VATS procedure with a transesophageal echocardiograph used during the entire case. This revealed dense lung adhesions to the lateral chest wall and a small right-sided pleural effusion. A portion of the pericardium was excised, and 150 mL of thick pericardial fluid was aspirated and sent for cytology. PcE was loculated and very dense, making it extremely challenging to remove (Figures 1B, 1C). Due to adhesions and poor visualization, further attempts to drain the pericardium were aborted. In efforts to avoid repeated pericardial drainage, a PleurX catheter was placed and looped in, limited by adhesions and loculations. Final pathology revealed reactive changes without malignancy.

While initially promising, minimal drainage was observed over time, leading to the removal of the catheter a month later. The patient was evaluated for pericardiectomy but was not considered a candidate due to the absence of cardiac tamponade symptoms and high risk of complications. She has continued to have a tortuous clinical course requiring frequent admissions to the hospital due to aspiration pneumonia and recurrent pleural effusions requiring chest tube placements. The palliative care team is involved to focus on the patient’s quality of life.

## Discussion

This case underscores the complexity of managing recurrent PcEs in patients with challenging anatomy and significant comorbidities. Current guidelines recommend a stepwise approach to PcE management: observation for small, asymptomatic effusions; pericardiocentesis for larger or symptomatic collections; and surgical window or pericardiectomy for recurrent or refractory cases [3]. These strategies, however, may be insufficient in patients who are poor surgical candidates, recurrence, or whose anatomy precludes effective drainage.

PiCs are well established in the management of malignant pleural effusions and ascites, providing both palliation and the potential for spontaneous pleurodesis [5]. Their role in PcE remains far less clear. From a technical standpoint, catheter placement in the pericardium presents additional challenges compared to the pleural space. The pericardial cavity is small, constrained, and closely associated with critical cardiac structures, with loculations that can further compartmentalize this limited space.

To date, only two published cases describe the use of PiCs in the pericardial space (Table 1). Louka et al. reported successful management of recurrent PcE in a heart transplant recipient, while Pustelnik et al. described durable palliation in a patient with malignant PcE [6,7]. In both cases, the effusions were free-flowing, allowing for continuous drainage and eventual catheter removal.

**Table 1:** Clinical summary of three reported cases of pericardial PiC. HT = heart transplantation; PCC = pericardiocentesis; PcE = pericardial effusion; PiC = permanent indwelling catheter; PW = pericardial window; s/p = status post; US = ultrasound; VATS = video-assisted thoracic surgery

Author/Year	Patient	Etiology of PcE	Prior interventions	PiC placement approach	Outcome
Louka et al. (2016) [5]	51-year-old female	Transudative PcE s/p HT	Multiple PCCs, limited thoracotomy w/ PW, corticosteroids, colchicine	PiC placed pericardially	Free-flowing, successful; PiC drained daily until output dropped to zero; catheter removed after 37 days; no recurrence on follow-up
Pustelnik et al. (2022) [6]	69-year-old male	Malignant PcE, secondary to disseminated lung adenocarcinoma	Multiple PCCs, thoracoscopic PW	PiC inserted via existing drain under US guidance	Free-flowing, successful; PiC resulted in 200 mL drainage after 9 days; catheter removed after 93 days; no recurrence
Our case	36-year-old female	Chronic inflammatory/idiopathic PcE	Failed PCC, failed surgical PW	VATS PW with tunneled PiC placement	Loculated, unsuccessful; minimal PiC drainage due to loculations; catheter removed after 1 month; recurrence of effusions; managed palliatively

In contrast, our patient’s effusion was heavily loculated and complicated by dense adhesions. Despite appropriate positioning, the catheter produced persistently minimal drainage, resulting in early catheter removal. This distinction highlights an important limitation: the success of indwelling catheters in the pericardium may depend less on the device itself and more on the characteristics of the effusion. Free-flowing or malignant effusions may be amenable to continuous drainage, whereas loculated or inflammatory effusions may resist catheter-based management.

It should be noted that intrapleural fibrinolytic agents, including tissue plasminogen activator, urokinase, and streptokinase, are instilled via PiCs to improve drainage and relieve symptoms when loculations or poor catheter function occur [8-10]. This approach is supported by multicenter observational studies and systematic reviews, which demonstrate that intrapleural fibrinolytic therapy can increase pleural fluid drainage and improve dyspnea in patients with PiCs complicated by loculations, with a generally acceptable safety profile and a low but present risk of pleural bleeding. Analogous to intrapleural fibrinolytic therapy for pleural effusion, intrapericardial fibrinolytics may hold potential in cases complicated by loculations. In our case, the absence of such adjunctive therapy likely contributed to the limited drainage and ultimate removal of the PiC placement. Furthermore, intrapericardial fibrinolytics were not considered given that use in the pericardial space is not established, carries a bleeding risk, and is unlikely to be effective in sthe etting of dense adhesions and thick, gelatinous loculations.

Beyond technical considerations, this case emphasizes the importance of multidisciplinary decision-making and the integration of palliative care in complex patients. In our patient, repeated interventions had already failed, pericardiectomy was deemed too high-risk, and the use of a PiC represented a last-resort effort to palliate recurrent effusions. While unsuccessful, the attempt illustrates the willingness of the care team to exhaust all options in pursuit of symptom relief and aligns with patient-centered care principles.

## Conclusions

This case adds to the very limited literature on PiC use for PcEs. While prior reports have demonstrated success in malignant or free-flowing effusions, our experience underscores that PiCs are unlikely to be effective in the setting of dense adhesions and loculations. As a single-case report involving an off-label intervention with only sparse, sometimes abstract-level, published comparators, these observations should be interpreted as hypothesis-generating rather than definitive. Our findings suggest that PiCs may be more suitable for free-flowing effusions and less effective in loculated or adhesive pericardial spaces. This emphasizes the importance of careful patient selection and multidisciplinary decision-making. Future research should aim to refine selection criteria, establish multicenter registries for outcome reporting, and explore adjunctive strategies such as intrapericardial fibrinolytics or catheter design modifications. Given the rarity of this clinical scenario, our case being only the third documented in the literature, prospective randomized trials are unlikely to be feasible. Nonetheless, reporting both successes and failures is essential to better define the potential role, limitations, and risks of PiC use in PcE management.
